# Combined Effects of Yellow Mealworm (*Tenebrio molitor*) and *Saccharomyces cerevisiae* on the Growth Performance, Feed Utilization Intestinal Health, and Blood Biomarkers of Nile Tilapia (*Oreochromis niloticus*) Fed Fish Meal-Free Diets

**DOI:** 10.1007/s12602-023-10199-8

**Published:** 2023-12-29

**Authors:** Ehab M. Anany, Mostafa A. Ibrahim, Ibrahim M. Abd El-Razek, El-Said M. El-Nabawy, Asem A. Amer, Amr I. Zaineldin, Mahmoud S. Gewaily, Mahmoud A. O. Dawood

**Affiliations:** 1 Animal Production Department, Faculty of Agriculture, Kafrelsheikh University, Kafr El-Sheikh, Egypt; 2 Department of Entomology, Faculty of Agriculture, Kafrelsheikh University, Kafr El-Sheikh, Egypt; 3 Department of Fish Nutrition, Central Laboratory for Aquaculture Research (CLAR), Agricultural Research Center (ARC), Abbassa, Giza, Egypt; 4 Agriculture Research Center, Animal Health Research Institute (AHRI-DOKI), Giza, Egypt; 5 Department of Anatomy and Embryology, Faculty of Veterinary Medicine, Kafrelsheikh University, Kafr El-Sheikh, Egypt; 6 The Center for Applied Research On the Environment and Sustainability, The American University in Cairo, Cairo, 11835 Egypt

**Keywords:** Aquafeed, Histology, Immunity, Insect meal, Probiotics

## Abstract

Aquafeed quality is the most critical factor for aquaculture sustainability. However, limitations of traditional feed ingredients such as fishmeal (FM) need alternative strategies to ensure the nutritional requirements for aquatic animals. In this trial, four test diets were formulated (2 × 2 factorial design), where FM was incorporated in two diets at 10% with or without *Saccharomyces cerevisiae* (SC) at 1 g/kg. At the same time, FM was replaced with yellow mealworm (*Tenebrio molitor*) meal (TM) with or without SC at 1 g/kg. The growth performance indices (final weight, weight gain, and SGR), and the feed utilization indices (FCR and PER) were markedly affected by the protein source (FM or TM) and dietary SC (*P* < 0.05). The protein source (FM or TM) significantly (*P* < 0.05) affected the whole-body protein and lipid contents, while the moisture and ash contents were unaffected (*P* > 0.05) by TM or SC. The growth of the intestinal villi showed a marked increase in both height and branching in the treated groups with SC along the whole length of the intestine. Furthermore, the immune cell infiltration was prominent near the intestinal crypts of the middle intestinal segments in the supplemented groups by SC. Dietary TM and SC revealed improved hepatic parenchyma in the liver tissue better than other groups. The hematological indices, including hemoglobulin, hematocrit, red blood cells, and white blood cells, were markedly affected by dietary SC (*P* < 0.05). The lysozyme activity and phagocytic index were markedly affected by dietary SC, while phagocytic activity was affected by dietary TM (*P* < 0.05). The catalase, glutathione peroxidase, and malondialdehyde were markedly affected by the interaction between dietary protein source and SC, while superoxide dismutase was affected by dietary SC (*P* < 0.05). In conclusion, adding SC could enhance the utilization of TM by Nile tilapia with positive effects on the intestinal and liver histological features and the immune and antioxidative responses.

## Introduction

Aquaculture is a vital activity for providing healthy seafood and livelihood for humanity [1–3]. Several fish species have contributed to farming and presented suitable and feasible solutions worldwide [4]. Carps, salmonoids, and tilapia are the most cultured fish species globally and attracted attention due to their high quality, demand, and commercial value [5, 6]. More specifically, Nile tilapia (*Oreochromis niloticus*), which is known as “aquatic chicken,” is considered the third most consumed fish species [7]. Nile tilapia offers a high potential to grow under diverse environmental conditions [8]. Besides, it has the ability to grow in intensive systems with a relatively lower cost of production than other farmed fish species [9]. Like other fish species, Nile tilapia require specific amounts of protein that mainly depend on fishmeal (FM), plant proteins, and food byproducts [10, 11]. However, the high prices of these ingredients, especially FM, and the unavailability and high demand for formulating carnivorous fish species feed limited the expansion of Nile tilapia production [12]. Therefore, replacing traditional ingredients with non-traditional nutritional components is crucial for Nile tilapia sustainability [12, 13]. Indeed, FM is a rich source of animal protein, essential amino acids, and flavors; thus, comparable protein sources may offer suitable replacers in Nile tilapia feeds [14, 15].

Recently, circular bio-economy initiatives were implemented to utilize the ever-increasing waste from different human activities [16–19]. In this regard, insect meal was introduced as an alternative nutritional and cost-effective ingredient for the sustainable aquafeed industry [11, 20–22]. More specifically, yellow mealworm (*Tenebrio molitor*) meal (TM) has been introduced to the aquafeed industry as a friendly environmental strategy to convert food waste into high-value feed ingredients [23]. The meal of TM is a rich source of protein required for formulating nutritionally balanced aquafeed [24]. The protein content of TM is comparable to FM, which is about 60%, and, therefore, could successfully replace FM [25]. Additionally, dietary TM contains chitin and riboflavin involved in various immunological, antioxidative, and antibacterial effects [26, 27]. Therefore, the inclusion of TM was investigated in several fish species as a partial or complete replacer for FM or soybean meal [28, 29]. However, the high chitin content in TM would interrupt the digestibility of nutrients in fish intestines, leading to lowered feed utilization and growth performance [30]. Hence, introducing functional additives such as medicinal herbs and probiotics may protect from the negative impacts and end with positive roles on fish intestines [31–34].

Undoubtedly, live beneficial microorganisms such as bacteria, fungi, and yeast were proven to enhance aquatic animals' intestinal health and feed digestion [7]. Yeast supplementation has been notably applied in aquaculture as a nutritional and bioactive additive [35]. More specifically, *Saccharomyces cerevisiae*, which has been used widely in aquaculture, is associated with its high protein content, growth-promoting, immunomodulation, and health benefits [36]. In addition, *S. cerevisiae* contains functional components in its cell walls, such as polysaccharides, mannan oligosaccharides, β-glucan, and chitin, which activate the entire body’s immunity [37, 38]. In this context, *S. cerevisiae* can enhance intestinal health through colonization in the mucosal layer, competition with harmful invaders, and inhibiting pathogens [39]. Consequently, *S. cerevisiae* can protect the intestinal epithelium health and regulate intestinal digestion, physiological function, and immunity [40].

In Nile tilapia, dietary *S. cerevisiae* enhanced the digestion capacity, intestinal health, immunity, and resistance to harmful pathogens [41–43]. In addition, dietary TM was included in tilapia diets without negative impacts [28]. However, the combined beneficial effects of TM and *S. cerevisiae* still need to be investigated. Thus, this study tested the combined effects of TM and *S. cerevisiae* on the growth performance, feed utilization, intestinal health, and blood biomarkers of Nile tilapia-fed FM-free diets.

## Materials and Methods

### Design and Diet Formulation

The trial was conducted at the laboratories and greenhouses for the Faculties of Agriculture and Veterinary Medicine, Kafrelsheikh University, Egypt. Uniform-sized Nile tilapia juveniles were obtained from a private farm located at the international road to Baltim city, Kafrelsheikh, Egypt, and gently transported to the wet laboratory. Carefully, all fish were randomly distributed and stocked in 15 well-cleaned and prepared glass aquaria (100 L) for acclimatization. All aquaria were equipped with electrical aerators and dechlorinated water, which was exchanged at 50% daily. Fish was offered the basal diet at 3% for 15 days, and the remaining feed was siphoned immediately. Subsequently, all fish were redistributed in the glass aquaria at 20 fish per aquarium with an average initial weight of 6.04 ± 0.05 g/fish. The trial was done in four groups, with three glass aquaria replicates representing each group. Every three aquaria were subjected to one of the test diets. Fish were offered the test diets twice daily at 08:00 a.m. and 2:00 p.m., and the amount of feed consumed was recorded. The feed intake was offered until satiation, and when the fish rejected the feed, the feeding was stopped. Feed was offered slowly to avoid excessive feed addition and to calculate the feed intake for each aquarium accurately. The water was regularly exchanged, and feces were siphoned in each aquarium during the 60-day feeding trial. The water quality was checked using the laboratory apparatus and recorded: 27.63 ± 0.29 °C; 7.42 ± 0.28; 6.08 ± 0.23 mg/L; and 0.01 ± 0.001 g/L for temperature, pH, dissolved oxygen, and total ammonia nitrogen, respectively. Fish were kept in 12-h light and 12-h dark intervals during the trial.

According to the NRC [44] and Hassaan and Mohammady [45], four test diets were formulated to meet the needs of Nile tilapia (Table 1). Four test diets were formulated (2 × 2 factorial design), where FM was incorporated in two diets at 10% with or without *Saccharomyces cerevisiae* (SC) at 1 g/kg. At the same time, FM was replaced with yellow mealworm (*Tenebrio molitor*) meal (TM) with or without SC at 1 g/kg. The SC (10 × 10^9^ cells/g) was produced by Levitan (Dox-al Italia S.P.A., Italy) and added to the diets at 1 g/kg, according to the recommendation of Abdel-Tawwab and Abdel-Rahman [46]. TM meal was prepared at the Department of Entomology, Faculty of Agriculture, Kafrelsheikh University, and its chemical composition was checked by following the AOAC [47]. The chemical composition (% of dry matter) for TM is 55.82%, 32.24%, 5.42%, and 6.32% for crude protein, total lipids, ash, and fibers, respectively. All ingredients were finely grounded and were thoroughly well mixed using the laboratory food mixer. Water was added to the ingredients at 35–40% to the dry mix for having a dough and then pelleted using the laboratory pelletizer. The pellets were kept at 2 mm size and were air dried inside the laboratory to avoid any possible accumulation threats; then, pellets were appropriately broken to keep suitable sizes for fish. After completely drying, the pellets were collected and kept in plastic bags and then stored in a freezer at − 20 °C. The chemical composition of the test diets was checked by following the AOAC [47]. The actual number of SC in the diets was confirmed by culturing the dilutions on YPD agar plates (2% peptone, 2% glucose, 1% yeast extract, and 1.5% agar) [48].

**Table 1:** Formulation and composition of the basal diet

Ingredients (g/kg)	FM	FM/SC	TM	TM/SC
Fish meal (FM) (65% cp)	100	100	0	0
*Tenebrio molitor* meal (TM)^1^	0	0	100	100
Soybean meal (44% cp)	320	320	330	330
*Saccharomyces cerevisiae* (SC)	0	1	0	1
Yellow corn	180	180	180	180
Corn gluten	60	60	60	60
Wheat bran	180	180	180	180
Rice bran	70	70	70	70
Wheat flour	52.5	51.5	52.43	51.43
Fish oil	20	20	10	10
Corn oil	10	10	10	10
Vitamin and mineral mix^2^	5	5	5	5
Dicalcium phosphate	2	2	2	2
L-methionine	0	0	0.02	0.02
L-lysine	0	0	0.05	0.05
Vitamin C	0.5	0.5	0.5	0.5
Total	1000	1000	1000	1000
Chemical composition				
Crude protein (%)	30.60	30.44	30.12	30.37
Crude lipids (%)	5.60	5.49	6.54	6.12
Ash (%)	5.11	5.21	5.21	2.19
Fibers (%)	4.62	4.96	4.62	4.67
Nitrogen-free extract (%)	54.07	53.90	53.51	56.65
Gross energy (MJ/kg)^3^	18.73	18.62	18.90	19.33
P/E ratio^4^	16.33	16.35	15.94	15.71

^1^The chemical composition (% of dry matter) for yellow mealworm (*Tenebrio molitor* (TM)) is 55.82%, 32.24%, 5.42%, and 6.32% for crude protein, total lipids, ash, and fibers, respectively.

^2^The mixture of vitamins and minerals is detailed by Hassaan et al.[45]

^3^Gross energy (GE) was calculated based on protein, lipid, and carbohydrate values as 23.6, 39.5, and 17.2 kJ/g, respectively[44]

^4^Protein to energy ratio (P/E) ratio (mg CP/kJ GE) = CP/GE × 1000 (kJ/100 g diet)

### Final Sampling

At the end of the trial, all fish were satiated for 24 h and then anesthetized with tricaine methane sulfonate (MS-222) (100 mg/L) to avoid handling stress. The individual weight and the total number of fish in each aquarium were recorded; then, randomly, three fish per aquarium were subjected to blood sampling and dissection. Samples for middle intestines and liver tissue from the four groups were dissected and fixed in 10% neutral buffered formalin.

Blood samples were collected from the caudal vein using 5 ml gauge syringes. The collected blood was then divided into two parts: half was stored in EDTA-heparinized tubes for immediate hematological analysis, and the other half was placed in non-heparinized tubes for serum collection. After 2 h, the blood samples in non-heparinized tubes were centrifuged at 1008 × g for 15 min at 4 °C. The serum was then separated and kept at − 20 °C for further analysis.

### Histology Study

After 24 h, the collected samples were transferred from 10% neutral buffered formalin to 70% alcohol. The intestine and liver samples were then dehydrated in ascending graded series of ethanol, cleared in xylene, and impregnated and embedded in paraffin wax by following Bancroft and Stevens [49]. Sections of 5 µm were cut using Leica rotatory microtome (RM 20352035; Leica Microsystems, Wetzlar, Germany) and mounted on glass slides. The prepared tissue sections were subjected to conventional staining of hematoxylin and eosin (H&E) according to Gewaily and Abumandour [50]. The stained sections were examined under a light microscope (Olympus, Tokyo, Japan).

### Blood Hematology

White blood cell (WBC) and red blood cell (RBC) counts and hemoglobin concentration (Hb) were done following standard procedure [51]. Hematocrit (Hct) was determined by the microhematocrit method while the hemoglobin (Hb) concentration was determined with a spectrophotometer (Model RA 1000, Technicon Corporation, USA) at 540 nm using the Blaxhall and Daisley [52] method.

### Blood Immunity and Antioxidative Status

A turbidimetric assay, based on the method of Ellis and Stolen [53], was used to analyze serum lysozyme activity. Leukocyte phagocytic function was assessed following the technique described by Cai and Li [54], where the percentage of leukocytes that engulfed bacteria was determined relative to the total leukocyte count in the smear. The phagocytic activity and index were determined as per Kawahara and Ueda [55].

To measure the levels of superoxide dismutase (SOD), catalase (CAT), and glutathione peroxidase (GPx), diagnostic reagent kits from Cusabio Biotech Co., Ltd. (China) were used as per the manufacturer’s instructions. Malondialdehyde (MDA) concentration was detected following the advice of Uchiyama and Mihara [56] and expressed in nmol MDA/mL.

### Calculations and Statistical Analysis

The following equations were used to calculate the weight gain (WG), specific growth rate (SGR), and survival rate. Besides, the feed conversion ratio (FCR), the protein efficiency ratio (PER), and feed intake were determined.

$$\mathrm{WG}=100\times(\mathrm{final}\;\mathrm{weight}\;(\mathrm{FW},\;\mathrm g)-\mathrm{initial}\;\mathrm{weight}\;(\mathrm{IW},\;\mathrm g))/\mathrm{IW}\;(\mathrm g)$$

$$\mathrm{SGR}\;(\%/\mathrm{day})=100\;\times\;(\ln\;\mathrm{FW}\;(\mathrm g)-\ln\;\mathrm{IW}\;(\mathrm g))/\mathrm{days}$$

$$\mathrm{FCR}=\mathrm{total}\;\mathrm{dry}\;\mathrm{feed}\;\mathrm{intake}\;(\mathrm g)/\;(\mathrm{FW}\;(\mathrm g)-\mathrm{IW}\;(\mathrm g))$$

$$\mathrm{PER}=(\mathrm{FW}\;(\mathrm g)-\mathrm{IW}\;(\mathrm g))/\mathrm{dry}\;\mathrm{protein}\;\mathrm{intake}\;(\mathrm g)$$

$$\mathrm{Survival}\;(\%)=100\times\mathrm{final}\;\mathrm{fish}\;\mathrm{number}/\mathrm{initial}\;\mathrm{fish}\;\mathrm{number}$$

All data were tested for homogeneity of variance by the Shapiro–Wilk and Levene tests, and normality was tested by the Kolmogorov–Smirnov test. Data were analyzed as a two-way ANOVA (2 factorial design) using the general linear model (GLM) procedure, the main protein sources (FM or TM), dietary SC, and their interaction. Tukey’s multiple comparison test compared means when interactive effects differed significantly. Values have presented an average of three replicates. Significant differences (*P* < 0.05) between dietary protein sources (FM or TM) and dietary SC were evaluated by Tukey’s test. All the statistical analyses were done via SPSS version 22 (SPSS Inc., IL, USA).

## Results

### Growth Performance

The growth performance indices, including final weight (FW), weight gain (WG), and SGR, and the feed utilization indices, including FCR and PER, were markedly affected by the protein source (FM or TM) and dietary SC (*P* < 0.05) (Table 2). The FW and WG were significantly higher in Nile tilapia-fed dietary FM with SC supplementation than those fed the other diets. In addition, the WG was significantly lower in Nile tilapia-fed TM without SC supplementation than in the other groups. The SGR was significantly higher in fish-fed FM and SC than in fish-fed TM without SC, without significant differences with the other groups. On the other hand, the FCR was significantly higher in Nile tilapia-fed TM without SC supplementation than in the other groups, while fish fed both FM and SC showed the lowest FCR value. The PER was significantly lower in Nile tilapia-fed TM without SC supplementation than in the other groups, while fish fed both FM and SC showed the highest PER value. The survival rate was not impacted (*P* > 0.05) by dietary TM or SC and was recorded at 95 to 98.33%.

**Table 2:** Growth performance of Nile tilapia fed test diets for 60 days

	IBW (g)	FBW (g)	WG (%)	SGR (%/day)	FCR	PER	Survival (%)
FM	6.03 ± 0.04	21.95 ± 0.33b	263.79 ± 4.09b	2.15 ± 0.02ab	1.41 ± 0.03b	2.33 ± 0.04b	98.33 ± 1.67
FM/SC	6.02 ± 0.05	23.87 ± 0.37a	296.71 ± 7.24a	2.30 ± 0.03a	1.27 ± 0.05c	2.58 ± 0.11a	96.67 ± 1.67
TM	6.02 ± 0.02	20.63 ± 0.22b	242.79 ± 2.82c	2.05 ± 0.01b	1.54 ± 0.03a	2.12 ± 0.05c	96.67 ± 1.67
TM/SC	6.05 ± 0.08	21.57 ± 0.13b	256.64 ± 6.66b	2.12 ± 0.03ab	1.45 ± 0.03b	2.26 ± 0.05b	95.00 ± 2.89
Two-way ANOVA (*P*-value)							
Protein source	0.860	0.000	0.001	0.000	0.003	0.005	0.438
SC	0.860	0.001	0.003	0.003	0.012	0.020	0.438
Protein source × SC	0.599	0.003	0.021	0.042	0.503	0.447	1.000

Values are an average of three replicates. Different letters indicate significant differences (*P* < 0.05) between dietary protein sources (fish meal (FM) or *Tenebrio molitor* meal (TM)) and *Saccharomyces cerevisiae* (SC) by Tukey’s test when significant interactions are seen at (*P* < 0.05). *IBW*, initial body weight; *FBW*, final body weight; *WG*, weight gain; *SGR*, specific growth rate; *FCR*, feed conversion ratio; *PER*, protein efficiency ratio

### Body Chemical Composition

The protein source (FM or TM) significantly (*P* < 0.05) affected the whole-body protein and lipid contents, while the moisture and ash contents were unaffected (*P* > 0.05) by TM or SC (Table 3). The total protein content was significantly lower in Nile tilapia-fed dietary TM without SC supplementation than those fed the other diets. Besides, the total lipid content was significantly higher in Nile tilapia-fed TM with SC supplementation than in the other groups.

**Table 3:** Body composition of Nile tilapia fed test diets for 60 days

	Moisture (%)	Crude protein (%)	Ether extract (%)	Ash (%)
FM	70.94 ± 0.06	18.19 ± 0.16a	5.26 ± 0.08b	5.27 ± 0.07
FM/SC	70.25 ± 0.24	18.76 ± 0.28a	5.57 ± 0.13b	5.33 ± 0.02
TM	71.06 ± 0.10	17.83 ± 0.06b	5.51 ± 0.09b	5.44 ± 0.03
TM/SC	70.53 ± 0.30	18.09 ± 0.29a	5.93 ± 0.15a	5.43 ± 0.16
Two-way ANOVA (*P*-value)				
Protein source	0.347	0.045	0.027	0.155
SC	0.115	0.093	0.012	0.795
Protein source × SC	0.691	0.505	0.651	0.711

Values are an average of three replicates. Different letters indicate significant differences (*P* < 0.05) between dietary protein sources (fish meal (FM) or *Tenebrio molitor* meal (TM)) and *Saccharomyces cerevisiae* (SC) by Tukey’s test when significant interactions are seen at (*P* < 0.05)

### Intestinal and Liver Histology

**Fig. 1:**
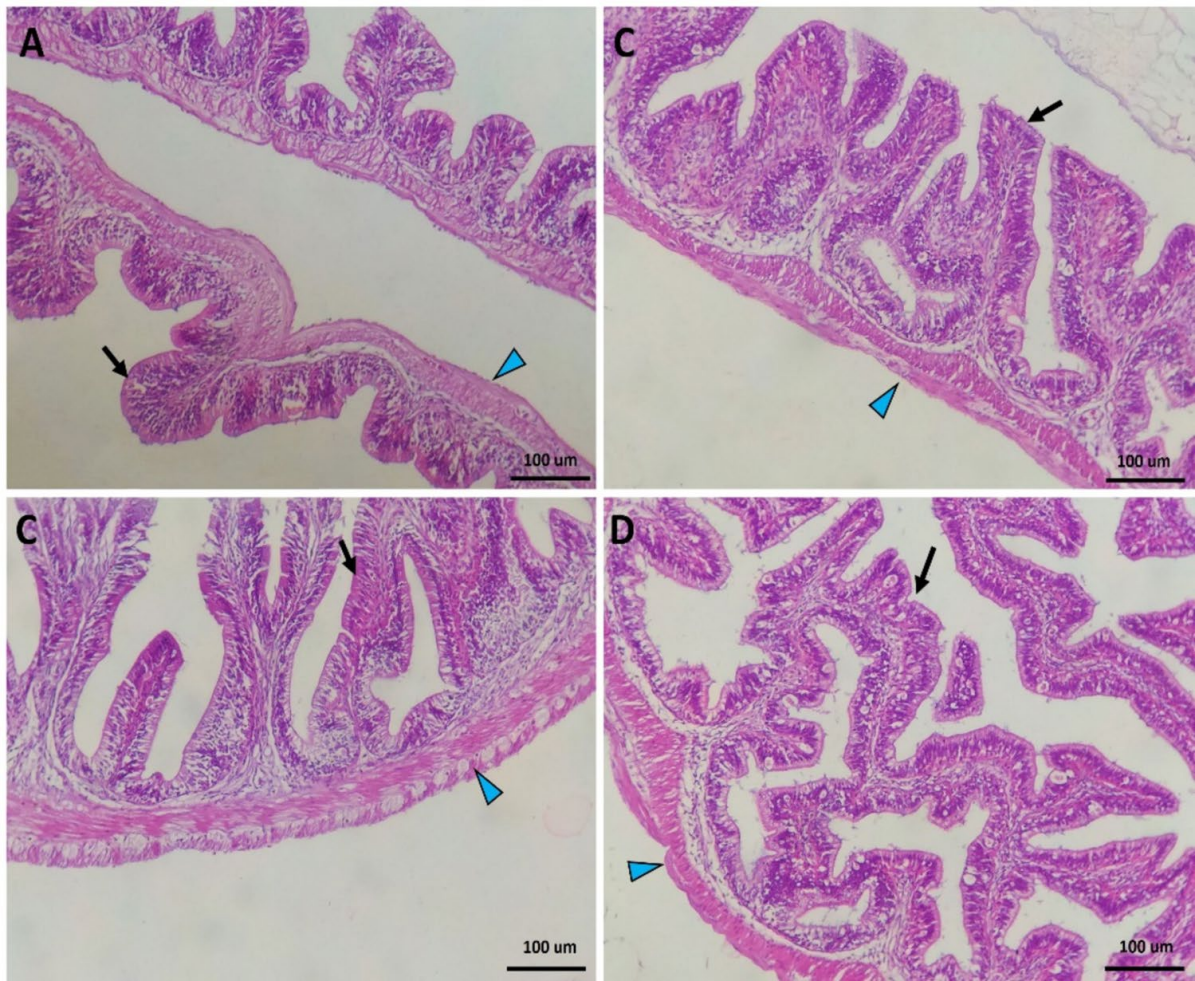
Histomicrograph showing the histological structure of middle segment of Nile tilapia intestine in the fish meal (FM) group (**A**) as well as other treated groups by *Tenebrio molitor* (TM) (**B**), *Saccharomyces cerevisiae* (SC) (**C**), and both TM with SC (**D**). The intestinal wall (IW) intestinal villi (IV) showed apparent growth mainly in group D in addition to immune cell infiltration (black arrow) in groups B and D. Stain H&E. Bar = 100 µm

**Fig. 2:**
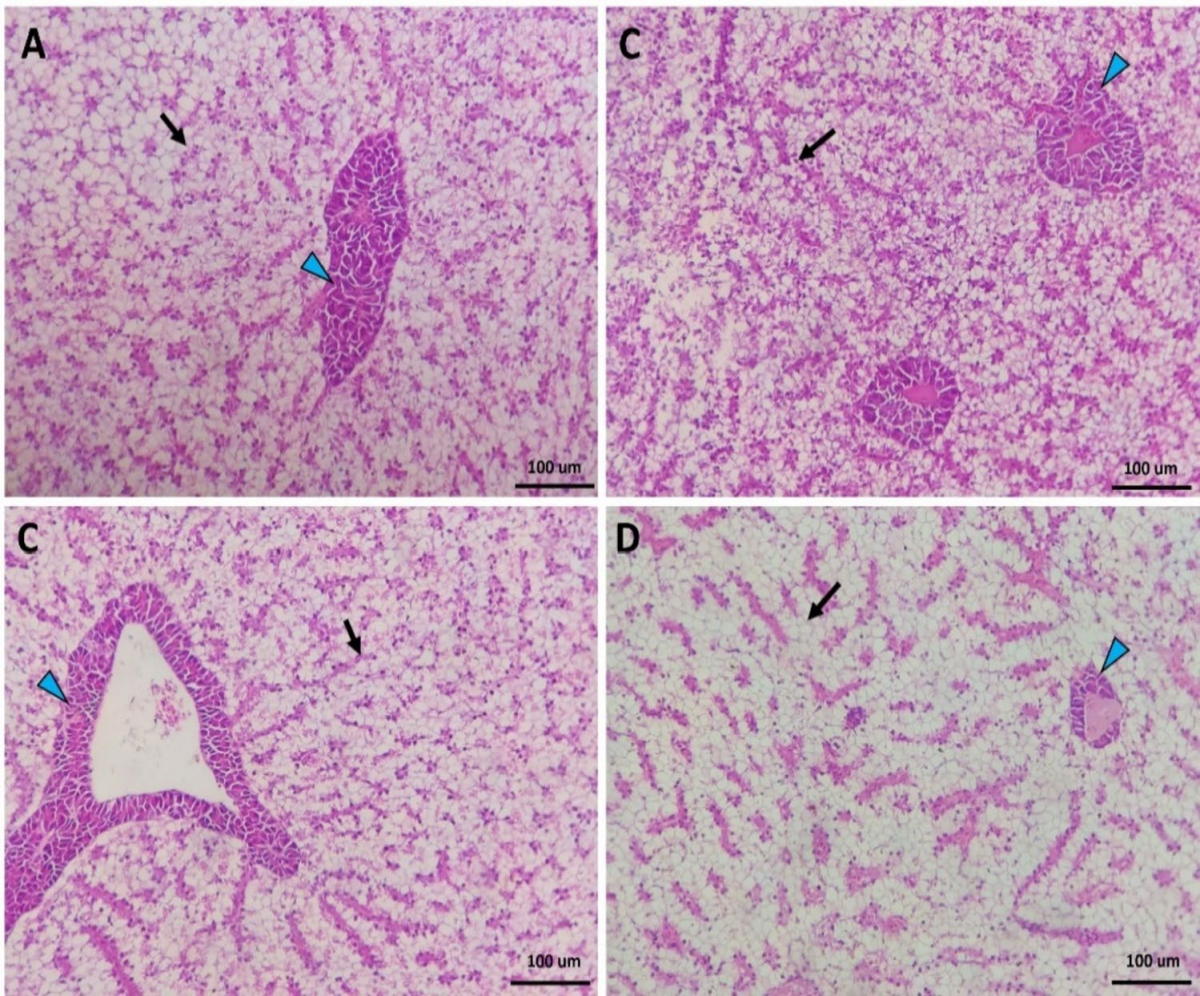
Histomicrograph showing the histological structure of hepatopancreas in the fish meal (FM) group (**A**) as well as other treated groups by *Tenebrio molitor* (TM) (**B**), *Saccharomyces cerevisiae* (SC) (**C**), and both TM with SC (**D**). The hepatic (H) and pancreatic (black arrow) structures in all groups appeared normal without any deterioration or vacuolation. However, the co-supplementation with both TM and SC stimulated severe glycogen deposition within the hepatocytes’ cytoplasm (**D**). Stain H&E. Bar 100 µm

The intestine of Nile tilapia showed intact structures of both intestinal walls and villi in all groups (Fig. 1A–D). The intestinal wall was formed of tunica mucosa internally, propria submucosa, and tunica muscularis, followed by serosa in the outermost layer. The intestinal villi projected within the lumen of the intestine were formed of simple columnar cells with goblet cells arranged around the connective tissue core. The growth of the intestinal villi showed a marked increase in both height and branching in the treated groups with SC along the whole length of the intestine (Fig. 1B–D). Furthermore, the immune cell infiltration was prominent near the intestinal crypts of the middle intestinal segments in the supplemented groups by SC (Fig. 2B, D).

The liver in the control fish revealed normal hepatic parenchyma, intact hepatocytes, and pancreatic acinar cells (Fig. 2A). Dietary SC-supplemented groups (Fig. 2B, C) showed similar histomorphology to the control group; however, the co-treatment with both TM and SC revealed improved hepatic parenchyma better than other groups. The hepatocytes showed increased glycogen deposition through irregular glycogen vacuoles inside their cytoplasm (Fig. 2D).

### Hematological Indices

The hematological indices, including hemoglobulin (Hb), hematocrit (Hct), red blood cells (RBCs), and white blood cells (WBCs), were markedly affected by dietary SC (*P* < 0.05) (Table 4). The Hb level was significantly higher in Nile tilapia-fed FM or TM with dietary SC than in fish-fed TM without SC. The RBCs were significantly higher in Nile tilapia-fed FM or TM with dietary SC than fish-fed FM without SC. The Hct level was significantly higher in Nile tilapia-fed FM with dietary SC than fish-fed FM without SC, without significant differences with fish-fed TM with or without SC (*P* > 0.05). The WBCs were significantly higher in Nile tilapia-fed FM or TM with dietary SC than in fish-fed FM or TM without SC.

**Table 4:** Hematological indices of Nile tilapia fed test diets for 60 days

	Hb (g/100 ml)	RBCs (10/mm^3^)	Hct (%)	WBCs (10/mm^6^)
FM	11.33 ± 0.51ab	3.56 ± 0.35b	37.33 ± 0.88b	9.96 ± 0.26b
FM/SC	12.58 ± 0.27a	4.21 ± 0.08a	40.00 ± 0.58a	10.95 ± 0.21a
TM	10.73 ± 0.25b	4.07 ± 0.09ab	39.00 ± 0.58ab	9.78 ± 0.09b
TM/SC	12.85 ± 0.22a	4.33 ± 0.03a	39.67 ± 0.88ab	10.24 ± 0.18a
Two-way ANOVA (*P*-value)				
Protein source	0.630	0.125	0.397	0.346
SC	0.001	0.040	0.046	0.043
Protein source × SC	0.226	0.328	0.217	0.346

Values are an average of three replicates. Different letters indicate significant differences (*P* < 0.05) between dietary protein sources (fish meal (FM) or *Tenebrio molitor* meal (TM)) and *Saccharomyces cerevisiae* (SC) by Tukey’s test when significant interactions are seen at (*P* < 0.05). *Hb*, hemoglobulin; *Hct*, hematocrit; *RBCs*, red blood cells; *WBCs*, white blood cells

### Immune Response

**Fig. 3:**
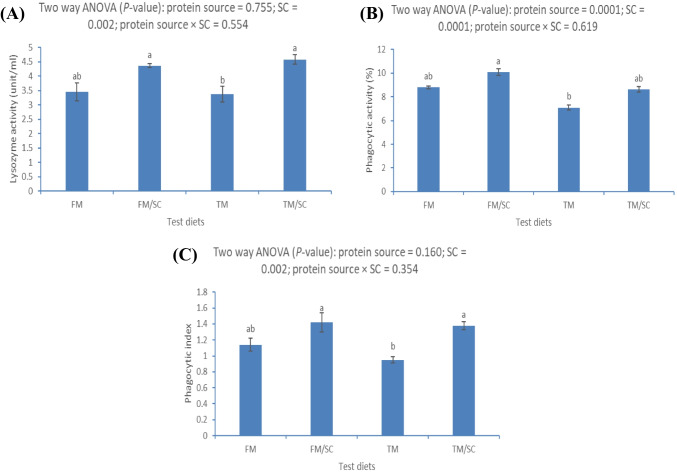
Blood immunity and antioxidative responses of Nile tilapia fed test diets for 60 days. Values are an average of three replicates. Different letters indicate significant differences (*P* < 0.05) between dietary protein sources (fish meal (FM) or *Tenebrio molitor* meal (TM)) and *Saccharomyces cerevisiae* (SC) by Tukey’s test when significant interactions are seen (*P* < 0.05)

The lysozyme activity and phagocytic index were markedly affected by dietary SC, while phagocytic activity was affected by dietary TM (*P* < 0.05) (Fig. 3). The lysozyme activity was significantly higher in Nile tilapia-fed FM or TM with dietary SC than in fish-fed TM without SC (Fig. 3A). The phagocytic activity was significantly higher in Nile tilapia-fed FM with dietary SC than in fish-fed FM without SC, without significant differences with the other groups (Fig. 3B). The phagocytic index was significantly higher in Nile tilapia-fed FM or TM with dietary SC than in fish-fed TM without SC (Fig. 3C).

### Antioxidative Response

**Fig. 4:**
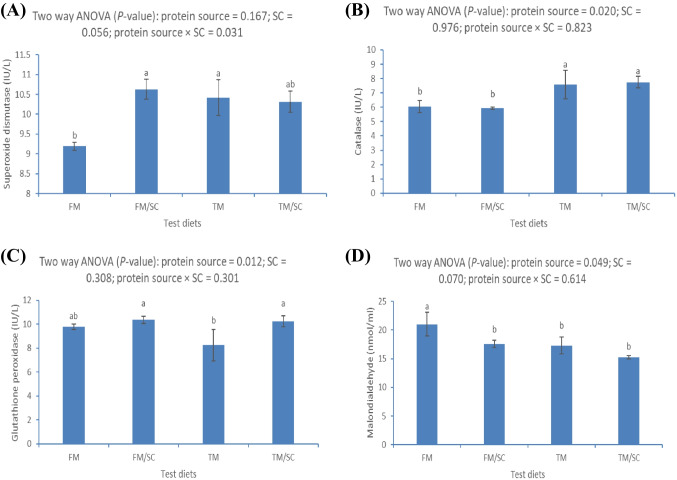
Blood antioxidative responses of Nile tilapia fed test diets for 60 days. Values are an average of three replicates. Different letters indicate significant differences (*P* < 0.05) between dietary protein sources (fish meal (FM) or *Tenebrio molitor* meal (TM)) and *Saccharomyces cerevisiae* (SC) by Tukey’s test when significant interactions are seen (*P* < 0.05)

The catalase (CAT), glutathione peroxidase (GPx), and malondialdehyde (MDA) were markedly affected by the interaction between dietary protein source and SC, while superoxide dismutase (SOD) was affected by dietary SC (*P* < 0.05) (Fig. 4). The SOD was significantly higher in Nile tilapia-fed FM with dietary SC or TM than in fish-fed FM without SC (Fig. 4A). The CAT was significantly higher in Nile tilapia-fed FM or TM with or without dietary SC than in fish-fed FM with or without SC (Fig. 4B). The GPx was significantly higher in Nile tilapia-fed FM or TM with dietary SC than in fish-fed TM without SC (Fig. 4C). However, the MDA was significantly lower in Nile tilapia-fed FM with SC or TM with or without SC than in fish-fed FM without SC.

## Discussion

Probiotic supplementation can enhance the utilization of non-traditional ingredients in aquafeed [7]. Mealworms such as *Tenebrio molitor* (TM) are safe and nutritious ingredients with high content of proteins, lipids, and minerals [57]. However, the high chitin component may impact intestinal health if TM is added at high levels [58]. In this consideration, *Saccharomyces cerevisiae* (SC) may enhance the utilization of TM-based feeds and limit the inclusion of unsustainable ingredients such as fishmeal (FM) [31, 32]. Dietary SC has been illustrated as efficient probiotics involved in regulating intestinal microbiota, mucosal integrity, and digestion capacity [35, 36]. The current study investigated the positive roles of dietary SC on Nile tilapia-fed TM as a replacer for FM.

Regarding the final weight, the results indicated that fish-fed FM and SC had shown the highest final weight. Besides, replacing FM with TM did not impact the final weight of Nile tilapia with or without SC addition. The results are similar to Tubin and Paiano [59], who could feed Nile tilapia with TM without remarkable effects on growth performance. The inclusion of SC also showed similar improvements in the growth performance of Nile tilapia, as stated by Abass and Obirikorang [43], Islam and Rohani [60], and Abdel-Tawwab [61]. The results of this study also indicated that the lowest weight gain and SGR were observed in Nile tilapia-fed TM without SC supplementation. Markedly fish-fed FM or TM with SC recorded high weight gain and SGR. As functional probiotics, dietary SC can enhance the growth performance of fish by enhancing feed utilization through (1) secretion of digestive enzymes (amylase, lipase, and protease) involved in feed degradation and digestion; (2) regulation of intestinal microbial balance through the inhibition of harmful pathogens and fortification of beneficial microorganisms, thereby protecting intestinal integration and absorption capacity [35, 36, 62]. On this occasion, the study also showed enhanced FCR and PER in the Nile tilapia-fed dietary SC in FM or TM-based diets. The improvements in the FCR and PER under the current trial conditions may explain the increased growth performance by dietary SC [43]. It is well-documented that probiotics can enhance the growth performance of aquatic animals and feed utilization by facilitating nutrient digestibility and absorption [60, 63].

In order to facilitate feed digestion and absorption, the intestine and liver histological features have to be considered [62]. Indeed, intestinal health status can always be evaluated by detecting morphological features, especially when testing new feed formulations in aquaculture [64]. Intestines are involved in feed digestion [65], while the liver is the metabolic organ involved in regulating physiological and metabolic functions [63, 66]. Exposure to toxic and harmful compounds that may be present in the feed interrupts liver function and induces hepatic failure [66, 67]. In the current investigation, the growth of the intestinal villi showed a marked increase in both height and branching in the treated groups with SC. Furthermore, the immune cell infiltration was prominent near the intestinal crypts of the middle intestinal segments in the supplemented groups by SC. Previous efforts showed similar results where dietary SC improved the intestinal villi length and width and enhanced the branching and mucosal integration in Nile tilapia [60], rohu (*Labeo rohita*) [68], and striped catfish (*Pangasianodon hypophthalmus*) [40]. A detailed explanation of how probiotics could improve the intestinal histological feature needs to be detailed. The high content of nucleotides in yeast walls could be involved in forming intestinal cell walls via the formation of RNA and DNA in the intestinal mucosa [69]. Therefore, the increased villi dimensions by dietary SC can increase the absorption area and effectively help feed utilization by fish. The results also indicated that the liver of fish fed SC revealed improved hepatic parenchyma better than other groups. The hepatocytes showed increased glycogen deposition through irregular glycogen vacuoles inside their cytoplasm. Similarly, Xia and Hao [63] illustrated that channel catfish (*Ictalurus punctatus*) fed dietary SC revealed improved liver histological features. Indeed, balanced feed formulations, the addition of probiotics, and the absence of xenobiotic hazards could be the main reasons for protecting liver health and, thereby, the entire body’s physiochemical function [7]. In this sense, dietary SC can overcome the negative impacts of high inclusion levels of TM. Besides, the healthy status of the liver confirms the safe use of TM with or without SC addition.

Whole body composition is a vital analysis associated with the effects of different feed ingredients and additives on the nutrient accumulation and weight gain of fish [70]. The results revealed that fish-fed TM showed the lowest protein content, while fish-fed TM and SC showed the highest lipid content. Similarly, Nile tilapia-fed dietary SC showed increased protein and lipid contents, as illustrated by Abdel-Tawwab and Abdel-Rahman [46]. The increased protein and lipid contents can be related to increased feed utilization by SC or FM, which results in a high accumulation of absorbed nutrients in the entire tissues of fish. The increased protein and lipid contents correspond to enhanced feed utilization (FCR and PER) and the growth performance results under the current trial conditions.

The study also exhibited meaningful effects on the hematological indices, which are tightly correlated with expressing fish's metabolic and physiological status [42]. The hemoglobulin, hematocrit, RBCs, and WBCs were enhanced by the addition of SC regardless of the protein source (FM or TM). Parallelly, Abdel-Tawwab and Abdel-Rahman [46], Jahan and Islam [68], and Boonanuntanasarn and Ditthab [40] reported enhanced hemoglobulin, hematocrit, RBCs, and WBCs in Nile tilapia, rohu (*Labeo rohita*), and striped catfish fed dietary SC. These hematological indices can be escalated due to the high yeast content from vitamin B complex and other hemotoxic substance involved in blood cell production [37, 38]. Additionally, increased WBCs by dietary SC can be related to enhanced immunity in Nile tilapia. Indeed, yeast cell walls contain abundant β-glucan (BG), which contains specific phagocytic cell receptors that contribute to WBC formation through binding with receptor molecules and release signals on the cell surface of the phagocyte [71].

The results also suggested that dietary SC enhanced the immunity of Nile tilapia-fed FM or TM. The immunity of Nile tilapia is evaluated by detecting lysozyme and phagocytic activities. Lysozyme activity is involved in damaging cellular walls for pathogenic bacteria [72], while phagocytic activity can limit the attack of harmful invaders [73]. Generally, probiotic supplementation is known for its role as an immunomodulator due to the direct intestinal immunity activation then mucosa and gut-associated lymphoid tissues [7, 74]. Similarly, dietary SC enhanced the lysozyme activity in Nile tilapia [41, 75] and striped catfish [40]. In addition, dietary SC improved phagocytic activity in Nile tilapia [41, 76], gilthead seabream (*Sparus aurata*) [77], and grouper (*Epinephelus coioides*) [76]. The production of inhibitory compounds by probiotics such as lactoferrin, lysozyme, bacteriocins, siderophores, and enzymes can directly inhibit pathogens [35]. Furthermore, yeast contains abundant functional substances such as BG and mannanoligosaccharides that immunomodulate the fish’s entire body [37, 38]. Activated phagocytic activity can also be correlated with increased WBCs by dietary SC, which is evident under the current study conditions.

Malnutrition and the absence of nutrients induce oxidative stress involved in the suppression of immunity, health, and growth performance of fish [62]. Herein, the current study considered evaluating the antioxidative status of Nile tilapia-fed FM or TM-based diets with or without SC. The results indicated marked improvement of the antioxidative responses (SOD, CAT, and GPx) while reducing the lipid peroxidation (low MDA) in Nile tilapia treated with SC. The results are in line with El-Nobi and Hassanin [75], who reported activated antioxidative responses in Nile tilapia-fed dietary SC. Enhanced antioxidative capacity can be related to the activation of the mucosal immunity and the entire body immunity of Nile tilapia-fed SC [78]. Further, the results indicated that Nile tilapia-fed TM showed a higher antioxidative response than those fed FM. Similarly, Zhang and Wu [28] and Sánchez-Muros and de Haro [79] reported that Nile tilapia-fed dietary TM revealed enhanced antioxidative capacity. The abundant chitin content in TM may be the direct reason for enhancing the antioxidative response via inhibiting lipid peroxidation and the formation of MDA [26, 27].

## Conclusion

To sum up, mealworms (*T. molitor*) can replace fishmeal in the diets of Nile tilapia without interrupting the growth performance and health status. Markedly, the addition of *S. cerevisiae* could enhance the utilization of TM by Nile tilapia with positive effects on the intestinal and liver histological features and the immune and antioxidative responses.

## Data Availability

All other relevant data are available from the corresponding authors upon reasonable request.
